# Mitigating bias in machine learning for medicine

**DOI:** 10.1038/s43856-021-00028-w

**Published:** 2021-08-23

**Authors:** Kerstin N. Vokinger, Stefan Feuerriegel, Aaron S. Kesselheim

**Affiliations:** 1 Institute of Law, University of Zurich, Zurich, Switzerland; 2 Program on Regulation, Therapeutics, and Law (PORTAL), Division of Pharmacoepidemiology and Pharmacoeconomics, Department of Medicine, Brigham and Women’s Hospital and Harvard Medical School, Boston, MA, USA; 3 ETH Zurich, Zurich, Switzerland; 4 LMU Munich, Munich, Germany

## Abstract

Several sources of bias can affect the performance of machine learning systems used in medicine and potentially impact clinical care. Here, we discuss solutions to mitigate bias across the different development steps of machine learning-based systems for medical applications.

Machine learning (ML) is an artificial intelligence technique that can be used to train algorithms to learn from and act on data^1^. ML in medicine aims to improve patient care by deriving new and relevant insights from the vast amount of data generated by individual patients and the collective experience of many patients^1,2^. The number of authorized ML-based systems has increased over the past years, with many being authorized by agencies such as the US Food and Drug Administration (FDA) for use in radiology, for example, to support tumor detection in diagnostic imaging^3^.

The creation of ML-based systems in medicine involves a number of steps. Relevant clinical data must first be collected and prepared for use in model development. Development of the model involves selection and training of suitable mathematical algorithms to perform the intended task. Model performance must subsequently be evaluated in independent cohorts of patients before potential authorization by agencies and deployment in clinical practice.

“The FDA has recognized challenges due to bias in ML and released an Action Plan in January 2021, highlighting the importance of identifying and mitigating bias in ML-based systems for medicine.”

It has been demonstrated that the outcomes of ML-based systems can be subject to systematic errors in their ability to classify subgroups of patients, estimate risk levels, or make predictions. These errors can be introduced across the various stages of development. Such errors are commonly referred to as bias^4–6^. For example, previous research has found that the application of a commercial prediction algorithm resulted in significant racial bias in predicting outcomes. Black patients assigned the same level of risk by the algorithm were sicker than white patients. This bias occurred because the algorithm used health costs as a proxy for health needs. Since less money is spent on black patients who have the same level of need, the algorithm falsely concluded that black patients were healthier than equally sick white patients^5^.

The FDA has recognized challenges due to bias in ML and released an Action Plan in January 2021, highlighting the importance of identifying and mitigating bias in ML-based systems for medicine^6^. Underrepresented groups in medical research are particularly susceptible to the impact of bias.

Previous studies have focused on the detection of bias^4^, but more discussion of possible solutions is needed. Here, we outline proposed solutions on how to mitigate bias across the different development steps of ML-based systems for medical applications (Fig. 1): data collection and data preparation, model development, model evaluation, and deployment in clinical practice (post-authorization).

## Data collection and data preparation

The first steps in which bias can be introduced are data collection and data preparation. In many ML-based applications in medicine, data of predictors, such as risk factors or other clinical parameters, serve as input, and, based on them, an outcome is predicted. For example, in the Framingham score, a tool to assess the cardiovascular risk of a patient, sociodemographics and other risk factors represent the predictors as input, while the risk level is the outcome that is to be predicted.

If the training data used to develop the ML-based system is subject to sampling bias, meaning that when the patient cohort in the data for training the ML model is not representative of the population for which the ML system is intended to be used, the same bias may be replicated when the system is applied in the clinical setting. For example, if a ML-based system is trained to recognize skin disease, such as melanoma, based on images from people with white skin, it might misinterpret images from patients with a darker skin tone, and might fail to diagnose melanoma^7^. This can lead to potentially serious consequences, since melanoma is responsible for the majority of skin cancer-associated deaths, and early diagnosis is critical for it to be curable^7^. To mitigate such bias, the developers of ML-based systems for medical applications should be transparent about the selected training data with regard to patient demographic and baseline characteristics, such as number of patients, distribution of patients’ age, representation of race and ethnicity, as well as gender. To address this type of bias, one should strive to compile datasets that are as diverse and large as possible to have a better representation of all patient groups. Developers can also carefully monitor error rates of ML software applied to different patient cohorts, and identify when the performance level deteriorates for a subset of patients. The performance level should then be disclosed by the developer in the authorization process.

In order to assess the risk of this type of bias, reporting checklists, such as PROBAST (prediction model risk of bias assessment tool), have been developed^8^. Such reporting checklists should be completed by developers and could guide the FDA in the authorization process to better understand the risk of potential bias of an ML-based system. Furthermore, it will also allow the end-users, such as physicians, to better understand whether the ML-based system is suitable in a specific setting for a specific group of patients.

## Model development

The modeling step uses mathematical algorithms to perform predictions, estimations or classifications in clinical parameters based on training data. The modeling step can perpetuate existing bias in the data. A naive application of a ML-based system without accounting for bias learns good predictions for the average population but does not necessarily incentivize the model to learn good predictions for those that are underrepresented in the data due to sampling bias (i.e., the underrepresented groups). In consequence, a model might perform overall better, yet it trades in a better performance for groups that are well represented at the cost of a lower performance (i.e., systematic errors) for the underrepresented groups.

Beyond creating diverse datasets for model development, there are mathematical approaches for de-biasing that mitigate the risk of bias at this step, such as adversarial de-biasing^9^ or oversampling^10^. Such approaches force the model to account for underrepresented groups and achieve a better performance when applied to them. However, techniques for de-biasing have only recently emerged in computer science and more research is still needed to demonstrate proof-of-principle and show that de-biasing reliably achieves its intended purpose. Systematic errors might also be reduced through continual learning^11^, whereby an ML-based system is continuously updated through new data while retaining previously learned knowledge^12^.

## Model evaluation

The model evaluation step, which is performed prior to authorization, is concerned with assessing how well the model makes predictions in independent groups of patients and in independent clinical studies and/or trials. This validates how the model generalizes to data from different patients and thus provides insights on how and where errors occur. Hence, it allows developers to identify bias that is introduced during the modeling step and also pinpoints to predictors that might be biased (e.g., due to a measurement error). Developers should carefully evaluate model performance, for example across certain subgroups of the patients, and inspect whether the model predicts incorrect outcomes.

Strategies to inspect how a ML model reaches an outcome can be grouped into techniques for interpretability or explainability^13^. Interpretability refers to models that are transparent in how outcomes are generated and where a user can understand the internal decision logic of the model (i.e., because the model has only a few parameters). By contrast, explainability means that a second model is created to explain the actual ML-based system, but where the actually ML-based system is not necessarily transparent (i.e., because it has millions of parameters). Explainability in ML is supported via various software tools, such as SHAP^14^ or LIME^15^. By understanding how a ML model reaches outcomes, developers can then validate the insights against prior knowledge from clinical research to ensure that a mathematical model considers known risk factors. In particular, developers may identify systematic errors in a ML-based system and then revise the model development accordingly, for instance by removing the responsible predictor or choosing a different model. However, caution would be recommended since ML explainability can be inaccurate or non-meaningful due to the underlying mathematical assumptions^13^. Hence, for high-risk ML-based systems in medicine, it might be better that models are limited to those that are interpretable.

## Deployment

In the deployment step, when the ML-based system has passed regulatory authorization and is implemented in clinical practice, bias can occur in situations where the patient cohort in clinical practice differs from the patient cohort in the training data, which is known as domain shift. This can lead to a deterioration in the performance with potentially negative outcomes for patients. Such a domain shift can, for example, occur if a ML-based system was developed with data from a US population, but is implemented in other geographies. To identify such unwanted bias, it is crucial that the ML-based systems are carefully monitored after authorization. This monitoring should include the following dimensions: the sociodemographics characteristics of patients to assess whether these are representative of the patients included in the training data; risk factors to check whether the patients have the same overall risk level because since, if the risk level differs, the ML model might no longer be precise; and the prediction performance of the ML-based system overall and across patients subgroups to identify other sources of error that were not known during model development. Monitoring ML-based systems after authorization is important to ensure that the performance does not degrade in clinical practice. If this occurs, the ML-based system needs to be updated with new post-authorization data.

Unwanted bias in clinical practice can also result from feedback loops. Feedback loops occur to when outcomes influence clinical practice so that a new bias is created. Post-authorization monitoring would also help identify such feedback loops so that steps can be taken to address its impact in clinical practice.

## Conclusion

Bias in ML-based systems for medical applications can occur across the different development steps, data collection and data preparation, model development, model evaluation, and post-authorization deployment in clinical practice. However, there are various strategies that reduce the risk of bias, including transparency about the selected training datasets, mathematical approaches to de-biasing, ML interpretability or explainability, and post-authorization monitoring. These strategies should become best practice for any ML-based system that is developed with medical application in mind. It is crucial that bias is mitigated when developing and deploying ML-based systems in medicine to prevent health care inequality for particular patient groups and to ensure a functionality that is safe for all patients.

## Figures and Tables

**Fig. 1:**
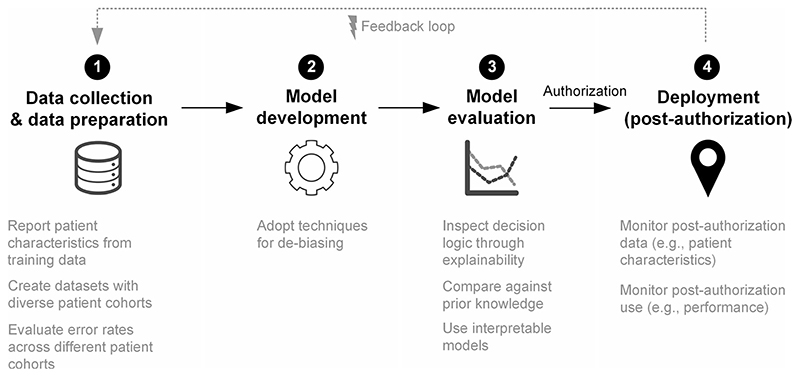
Strategies for mitigating bias across the different steps in machine learning systems development. Diagram outlining proposed solutions on how to mitigate bias across the different development steps of ML-based systems for medical applications: (1) Data collection and data preparation, (2) Model development, (3) Model evaluation, and (4) Deployment.

## References

[1] FDA (2021). Artificial intelligence and machine learning in software as a medical device 2021.

[2] Hwang TJ, Kesselheim AS, Vokinger KN (2019). Lifecycle regulation of artificial intelligence- and machine learning-based software devices in medicine. J Am Med Assoc.

[3] Muehlematter UJ, Daniore P, Vokinger KN (2021). Approval of artificial intelligence and machine learning-based medical devices in the USA and Europe (2015-20): a comparative analysis. Lancet Digit Health.

[4] Parikh RB, Teeple S, Navathe AS (2019). Addressing bias in artificial intelligence in health care. J Am Med Assoc.

[5] Obermeyer Z, Powers B, Vogeli C, Mullainathan S (2019). Dissecting racial bias in an algorithm used to manage the health of populations. Science.

[6] FDA (2021). Artificial intelligence/machine learning (AI/ML)-based software as a medical device (SaMD) action plan.

[7] Adamson AS, Smith A (2018). Machine learning and health care disparities in dermatology. JAMA Dermatol.

[8] Wolff RF (2019). PROBAST: a tool to assess the risk of bias and applicability of prediction model studies. Ann Intern Med.

[9] Zhang BH, Lemoine B, Mitchell M, Furman Jason, Marchant Gary, Price Huw, Rossi Francesca (2018). Mitigating unwanted biases with adversarial learning.

[10] Kamiran F, Calders T (2012). Data preprocessing techniques for classification without discrimination. Knowl Inf Syst.

[11] Vokinger KN, Feuerriegel S, Kesselheim AS (2021). Continual learning in medical devices: FDA’s action plan and beyond. Lancet Digit Health.

[12] Lee CS, Lee AY (2020). Clinical applications of continual learning machine learning. Lancet Digital Health.

[13] Rudin C (2019). Stop explaining black box machine learning models for high stakes decisions and use interpretable models instead. Nat Mach Intell.

[14] Lundberg S, Lee S-I (2017). A unified approach to interpreting model predictions.

[15] Ribeiro MT, Singh S, Guestrin C, Krishnapuram Balaji, Shah Mohak, Smola Alex, Aggarwal Charu, Shen Dou, Rastogi Rajeev (2016). “Why should i trust you?”: explaining the predictions of any classifier.

